# Neuronutrition and Its Impact on Post-Stroke Neurorehabilitation: Modulating Plasticity Through Diet

**DOI:** 10.3390/nu16213705

**Published:** 2024-10-30

**Authors:** Irene Ciancarelli, Giovanni Morone, Marco Iosa, Antonio Cerasa, Rocco Salvatore Calabrò, Maria Giuliana Tozzi Ciancarelli

**Affiliations:** 1 Department of Life, Health and Environmental Sciences, University of L’Aquila, 67100 L’Aquila, Italy; irene.ciancarelli@univaq.it (I.C.); mariagiuliana.tozzi@guest.univaq.it (M.G.T.C.); 2 ASL 1 Abruzzo (Avezzano-Sulmona-L’Aquila), 67100 L’Aquila, Italy; 3 San Raffaele Institute of Sulmona, 67039 Sulmona, Italy; 4 Department of Psychology, Sapienza University of Rome, 00185 Rome, Italy; marco.iosa@uniroma1.it; 5 IRCCS Fondazione Santa Lucia, 00179 Rome, Italy; 6 Institute of BioImaging and Complex Biological Systems (IBSBC-CNR), Via T. Campanella, 88100 Catanzaro, Italy; antonio.cerasa@cnr.it; 7 S. Anna Institute, 88900 Crotone, Italy; 8 IRCCS Centro Neurolesi Bonino Pulejo, 98124 Messina, Italy; roccos.calabro@irccsme.it

**Keywords:** stroke, rehabilitation, neuronutrition, oxidative stress, inflammation, gut microbiota, gut–brain axis

## Abstract

The recovery of neurological deficits after ischemic stroke largely depends on the brain’s ability to reorganize its undamaged neuronal circuits and neuronal plasticity phenomena. The consolidated evidence highlights the involvement of the patient’s impaired nutritional conditions in post-stroke recovery and unsatisfying rehabilitative outcomes. Standardized nutritional protocols usually applied in hospitalized patients in a rehabilitation setting aim mainly to improve the general health conditions of patients, do not consider the high inter-individual variability in neurorehabilitation outcomes, and are not sufficiently modifiable to provide neuroprotective and restorative dietary patterns that could promote neuronal plasticity and functional recovery during neurorehabilitation. Neuronutrition, an emergent scientific field of neuroscience, represents a valid model of a personalized nutritional approach, assuring, for each patient, nutrients having antioxidant and anti-inflammatory properties, ensuring a balanced microbiota composition, and providing adequate neurotrophic support, essential for improving neuronal plasticity, brain functional recovery, and rehabilitative outcomes. In the present narrative review, we provide an overview of the current knowledge on neuronutrition as an adjuvant strategy of a personalized nutritional approach potentially effective in improving post-stroke neuroplasticity and neurorehabilitation by counteracting or at least limiting post-stroke oxidative/nitrosative stress, neuroinflammation, and gut–brain axis disturbance.

## 1. Introduction

The recovery of neurological deficits after ischemic stroke, maximal in the subacute phase and much less effective in the chronic phase, largely depends on neuronal plasticity phenomena that trigger the brain’s capacity to reorganize its undamaged circuits through new connections, particularly increased in the cerebral tissue surrounding the necrotic area [1,2]. Parallel processes of the remodelling of neurovascular units start to allow connections among neurons and blood vessels, essential for providing nutritional substances to the brain [3,4,5]. Neurorehabilitation facilitates post-stroke brain reorganization and modulates neuronal excitability in damaged networks by developing neurobiologically guided therapies that exploit behavioural and neural signals to stimulate neuroplasticity [4,5,6,7]. Functional recovery after stroke and the effectiveness of rehabilitation treatment are strongly characterized by a high inter-individual variability dependent, at least in part, on neural signals driving plasticity that is modulated by genetic polymorphisms and epigenetic modifications of DNA that can “switch on” or “switch off” the gene-mediated response to internal or external bodily factors [8,9,10]. Moreover, different biological and clinical variables, pre-existing or resulting from ischemic brain damage, interfere with each other, thus influencing the rehabilitation response of each patient and the progress of the functional recovery [7,10,11]. Among the verifiable post-stroke complications, some of them could be avoided as reported and recommended by numerous stroke guidelines [7,12,13]. A poor nutritional status/malnutrition is one of the most frequent and common problems among stroke patients upon admission to hospital in a rehabilitation setting. The sequelae of the stroke such as dysphagia, hemiparesis, reduced mobility, sarcopenia, cognitive impairment/depression, and/or pre-existing stroke comorbidities, indeed, contribute to the deterioration of nutritional conditions also in patients not malnourished before the stroke’s occurrence, and impair the neurorehabilitation effectiveness [14,15,16]. Optimizing nutritional management and monitoring the nutritional status in all phases of stroke are, therefore, out of the question and among the urgent purposes in stroke care. Clinical evidence shows that the nutritional condition on admission predicts the rehabilitation outcomes. For patients with an adequate nutritional status at admission, there is insufficient evidence to support nutritional interventions [17], while they are strongly recommended for patients with malnutrition and for those at risk [17,18]. The link between the effectiveness of nutrition interventions for patients with an impaired nutritional status and rehabilitation effectiveness is also underlined by studies showing improved conventional outcome measures (the NIH Stroke Scale, the Barthel Index, the modified Rankin Scale, and the Functional Independence Measure), used to examine the neurological results and functional independence of patients after nutritional treatment [19,20,21,22,23]. Therefore, nutrition, due to its pleiotropic properties and in compliance with the neuroprotective and restorative needs required by the brain recovery process, is confirmed as a crucial exigency for post-stroke rehabilitative outcomes. Standardized nutritional protocols usually applied in hospitalized patients and in rehabilitation settings aim to improve mostly general health conditions of patients, namely, those with a poor nutritional status or with evident malnutrition, to reduce common clinical comorbidities, and avoid a delay in starting neurorehabilitation treatment and its decreased effectiveness. Nutritional interventions, in fact, often translates only into an increase in caloric and nutritional intake, various types of food supplementation, and a supplementation of minerals and vitamins, with particular emphasis on protein intake essential for correcting the protein–energy imbalance and counteracting muscle atrophy and sarcopenia [24,25,26,27]. In addition, homologated nutrition interventions, generally used for hospitalized patients, do not consider the high inter-individual variability in rehabilitation outcomes and are not changeable enough to provide dietary patterns that, owing to their specific neuroprotective properties, may favour neuroplasticity and, therefore, functional recovery during neurorehabilitation [17]. Oxidative stress and inflammation are strongly involved in the mechanisms underlying ischemic injury and repair processes following brain damage as well in the alterations in the nutritional status of stroke patients [5]. Based on this evidence, reinforced antioxidant and anti-inflammatory defence has been proposed as a strategy to facilitate brain recovery and improve the nutritional status. Accordingly, the experimental findings endorse the potential of this approach. Evidence from non-human studies suggests that docosahexaenoic acid (DHA) enhances the expression of brain-derived neurotrophic factor (BDNF) and provides a positive influence against tissue damage induced by brain injury [28]. Positive effects on neuroplasticity, neurogenesis, and cerebral blood flow were attributed to flavonoids, a family of compounds present in several edible plants with antioxidant and anti-inflammatory properties [29,30,31]. However, the translation of these experimental results into clinical practice still requires the definition of several aspects, and it is not conclusive [29]. There is now a great deal of interest in considering the conceptual framework of neuronutrition, an emerging scientific field of nutritional systems biology that places at the centre of its objectives the need for a personalized intervention and the consequent tailored dietetic program [5,32,33]. Technologies collectively called “-omics” enable the simultaneous measurement of an impressive number of biomolecules that can be potential markers for both the inter-individual capacity with which each patient responds to rehabilitation treatment, and the mechanisms with which dietary patterns are able to activate cellular signaling pathways involved in neuroplasticity [34,35]. Furthermore, the opportunity to empower patients to take an active role in their neurorehabilitative program, according to the principles of 5P medicine (predictive, preventive, participatory, personalized, and precision medicine) [36], could facilitate the adherence to consume a personalized diet based on the daily intake of nutrients promoting functional recovery and neurorehabilitation outcomes (Table 1). In this narrative review, we report the available evidence on the potential properties of some neuronutrients to influence positively the recovery processes and rehabilitative outcomes by interfering with post-stroke oxidative/nitrosative stress, neuroinflammation, and gut–brain axis disturbance.

## 2. General Overview on Functional Recovery Mechanism after Stroke

After cerebral ischemic injury, neural plasticity and brain tissue reorganization intervene in damaged and non-damaged areas of the brain. Cellular and molecular mechanisms with multiple underlying pathways, highly interconnected among themselves, are involved at restoring, replacing, and compensating brain-damaged structures and lost functions, albeit within certain limits, thus allowing neurorehabilitation training to use neural circuits modelled on new functional connections and the pruning of non-functional ones [11]. More understanding of the biological basis of this complex process is essential in order to develop new therapeutic approaches and rehabilitative strategies pivotal to improving functional recovery. As is known, the interruption of blood flow and the consequent deprivation of O_2_ and nutrients to the ischemic area lead to metabolic changes of the surrounding neurons, altered mitochondrial activity, the disruption of the blood–brain barrier, oedema, inflammation, and a strong imbalance of the vulnerable equilibrium between the generation of free radicals and the antioxidant defence [56,57,58]. The persistence after ischemic onset of the excessive generation and release of reactive oxygen species (ROS) and reactive nitrogen species (RNS), together with a long-lasting depletion of endogenous antioxidant defences, triggers cellular cytotoxic pathways responsible for neuronal necrosis and apoptosis, and remodelling cell–cell signalling, such as neurogenesis and angiogenesis [59]. The activation of transcription factors and proinflammatory gene expression results in an increased secretion of inflammatory cytokines [5,42]. Inflammation exacerbates the ischemic injury in the early phase and influences post-stroke recovery in the late phase, persisting for days and gradually fading away within weeks after the stroke’s onset. Microglia undergo activation and assume the role of one of the most important cellular components of post-stroke inflammation. Moreover, brain-resident cell-mediated inflammation represents the main source of oxidative stress after a stroke’s onset [5,42]. Oxidative stress and inflammation after stroke onset could represent, therefore, an interesting target to develop nutritional procedures as an adjuvant strategy to support post-stroke recovery and neuronal plasticity. Many bioactive molecules found in foods offer a potential positive influence on cerebral recovery as antioxidant and anti-inflammatory agents [35,60,61], even though their molecular structure, solubility, food matrix, and gut microbiota metabolism limit their bioavailability and absorption. Interestingly, efficacious technologies, now tested, may improve the above-mentioned limiting factors [62].

In concert with cerebral tissue repair and neuronal plasticity, vascular remodeling patterns occur. Angiogenesis is a protective tissue mechanism that promotes neural regeneration and functional recovery after stroke. Neurons and astrocytes express angiogenic factors to promote microvascular growth, and newly formed microvessels influence brain tissue repair via the release of growth factors such as BDNF [63,64,65,66,67,68]. In this scenario, the brain’s capacity for restoring lost functions and recovery proceeds spontaneously in the early period after stroke and then with compensatory neuronal plasticity based on the reorganization of neural circuits, the creation of new functional communications in the remaining neuronal circuits, and the enhancement of neuronal activity in pre-existing damaged networks [1,5]. Notably, the effectiveness of neuron plasticity depends on many factors including the patient’s clinical history, age, and education. Interestingly, evidence supports that there is a sort of brain cognitive reserve in skilled individuals that may have an impact on the recovery process [67]. Neurorehabilitation, as already reported, facilitates post-stroke brain reorganization and exerts a positive influence over ongoing neuronal plasticity. Of note, individual variability in response to rehabilitation treatment depends on different factors among which the patient’s nutritional status and clinical history are determinants. A rehabilomic approach, based on the analysis of candidate biomarkers expressing the mechanisms of functional recovery, may provide a valid integration of the patient’s clinical data to offer personalized nutritional interventions needed to optimize individual neurorehabilitation outcomes [69].

## 3. Neuronutrition as an Adjuvant Strategy in Post-Stroke Neurorehabilitation

Eating disabilities are frequent clinical complications that affect the nutritional status of patients after stroke, namely, aged individuals, and result in the development of unsatisfactory adherence to neurorehabilitation treatment because of stroke-associated sarcopenia with the consequent exacerbated loss of physical strength and fatigue, as well depressive behavioural aspects [70]. Moreover, the frequent aging- and stroke-induced impairment of taste and smell decreasing the physiologic pleasure of palatable foods further results in a decreased daily dietary intake [71]. Behind dysphagia, which is one of the most important complications following a stroke, the penalizing dependence on help for eating, together with disempowerment, boredom, frustration, and, in more serious cases, cognitive impairment, has, in fact, a detrimental impact on the proper quantity and composition of everyday food intake. The consequent nutritional deficiencies, specific or global, are crucial for the patient’s nutritional condition and negatively influence neurorehabilitation effectiveness [22,23,24]. The benefits of proper dietary patterns underline how the synergic interactions between diet components and the adequate intake of macronutrients, micronutrients, and bioactive substances contained in them are a fundamental requirement for maintaining one’s health status that, however, cannot be separated from a healthy lifestyle, especially regular physical activity [72,73]. Furthermore, the long-term consumption of balanced diets could prevent or attenuate the deterioration of cognitive functions and, possibly, the onset and progression of neurodegenerative diseases frequent, namely, in aging individuals [33]. The mechanisms associated with the benefits of proper dietary patterns are multiple, and include antioxidant and anti-inflammatory activity, the modulation of gut microbiota and gut functions, and increased neurotrophic support, as well as decreased neuronal damage [5]. Neuronutrition represents a valid model of a personalized nutritional approach toward optimizing neurological health, preventing neurological diseases, and favoring brain recovery in the event of damage, as occurs for neurodegenerative diseases and stroke [33,74]. Improving eating behavior through the correction of the patient’s diet altered by stroke and adopting dietary patterns with selected and appropriate foods with antioxidant and anti-inflammatory activity are essential in order to enhance post-stroke brain structural and functional recovery so as create clinical conditions favorable to rehabilitation outcomes. Oxidative/nitrosative stress, inflammation, gut–brain axis disturbance, and neurotransmitter imbalance are the more significant targets of neuronutrition [33,75,76,77]. According to the neuronutrition conceptual purpose, dietary patterns based on foods having bioactive components interfering with the main mechanisms induced by stroke onset could positively influence brain repair, and improve plasticity and functional recovery, thus favouring rehabilitative effectiveness.

## 4. Neuronutrition and Crosstalk Between Oxidative/Nitrosative Stress and Inflammation

Determining nutritional needs to support recovery and metabolic demands for post-stroke patients undergoing neurorehabilitation is difficult due to multifactorial conditions, including the altered patient feeding behaviour, altered levels of autonomic dysfunction, and hypercatabolic state, which interfere with brain recovery and all bodily repair processes [33,77,78,79,80]. Studies performed mainly on neurodegenerative diseases have emphasized the promising influence of a variety of foods, individual nutrients, and dietary habits on stemming or, at least, limiting oxidative/nitrosative stress and inflammation, as well as on strengthening antioxidant defences [74,75,76,77,78]. The hypothesis that nutrients or dietary patterns influencing the crosstalk between oxidative stress and inflammation could provide a neuroprotective influence on post-stroke recovery mechanisms and improve rehabilitative responsiveness remains of great interest. The relative findings are few, in fact, and there is no evidence, as far as we know, of the involvement of specific dietary patterns or single nutrients, present normally in foods, on rehabilitation outcomes because of their antioxidant and anti-inflammatory properties [27,57,81,82].

### 4.1. Oxidative/Nitrosative Stress and Inflammation

As is widely known, physiologic amounts of ROS and RNS are generated by all cells to act as signaling molecules, while the release of large amounts triggers or amplifies inflammation via an upregulation of different genes that code for proinflammatory cytokines and adhesion molecules working in the immune defense mechanism [83,84]. The consequent crosstalk between oxidative/nitrosative stress and inflammation is dynamically modulated by oxidative metabolites that activate inflammatory-signaling pathways, and by inflammatory cytokines that keep the oxidative stress “on” [5]. The development of brain-resident cell-mediated inflammation promotes the expansion of the ischemic lesion, blood–brain barrier dysfunction, and systemic inflammatory response that negatively affect the brain repair process [42,57,58,59]. A variety of enzymes and non-enzymatic molecules widely distributed in the intra- and extra-cellular compart constitutes the powerful antioxidant body’s system. Antioxidants, both endogenous and derived from diet, may mitigate the oxidative/nitrosative stress by removing potential oxidants or transforming them into less reactive compounds and tone down the cytokine storm and inflammation [77,78,84]. Superoxide dismutase (SOD), catalase, glutathione (GSH), GSH peroxidase (GPx), thioreductase, and uric acid are the main endogenous antioxidants. Of note, albumin, whose oxidized form is considered as a marker of oxidative stress, is actively involved in the redox status via its multiple-binding sites and free-radical-trapping properties that, overall, potentiate the bodily antioxidant status. The most relevant are connected to its ability to bind and transport in the systemic circulation bilirubin, polyunsaturated fatty acids, many dietary flavonoids, and polyvalent metal ions [85]. Main dietary sources of antioxidants include vitamins A, C, and E, as well as polyphenolic compounds and minerals. Folate and the B vitamins, crucial in the metabolism of methionine, exert a positive influence on antioxidant GSH, central to a variety of processes [44]. An excessive ROS amount induces the activation of the nuclear factor erythroid 2-related factor (Nrf2), a transcription factor recognizing the antioxidant response element that, in turn, increases the expression and activity of superoxide dismutase, glutathione peroxidases, and catalase, and reduces lipid peroxidation in brain tissue. Interestingly, the phytoalexin resveratrol exhibits a neuroprotective influence by inhibiting inflammation via the activation of the Nrf2 pathway [86,87]. Exogenous antioxidants such as polyphenols, polyunsaturated fatty acids (PUFAs), and vitamins A and C, as well as vitamin D could express neurorestorative effects essential for post-stroke recovery and rehabilitation effectiveness [27,42,43]. Therefore, antioxidant- and anti-inflammatory-rich foods could stop or modulate the stroke-associated oxidative and inflammatory chain reactions and provide a neuroprotective influence essential for enhancing the recovery process.

### 4.2. Role of Minerals in the Oxidative/Nitrosative Stress and Inflammation

The research findings showed the importance of the mineral intake in oxidative stress and inflammation, and mineral homeostasis might be a sensitive indicator of oxidative stress and inflammation in stroke patients [43,45,46,47,48,49]. Many studies underlined the essential role of some minerals such as zinc, magnesium, copper, and selenium, and their potential for antioxidant activity, not through the direct scavenging of free radicals, but instead through their requirement as regulatory co-factors in different antioxidant enzyme functions. The source of these elements is only foods of animal and vegetable origin, and the detrimental impact of eating disabilities on the everyday food intake of patients could account for their frequent deficiency. High levels of zinc (Zn) are present in the brain where it assumes multiples roles. Zinc deficiency results in weight loss and poor food efficiency [43,46]. The restoration of body weight, extremely important in assuring the effectiveness of rehabilitative interventions, should include adequate zinc in the diet [45]. Together with copper (Cu), Zn is a component of Cu/Zn superoxide dismutase that is responsible for scavenging superoxide anions and preventing nitric oxide decomposition at the vascular endothelium level [5]. Moreover, Zn absorption is reduced by a vitamin A deficiency [46]. Selenium (Se) is a dietary trace element that, in the form of selenoproteins, possesses remarkable antioxidant properties, immune functions, and metabolic homeostasis [47]. Glutathione peroxidase is a selenocysteine-dependent enzyme important for scavenging hydrogen peroxide and is strictly dependent on Cu/Zn and Cu/Se molar ratios and the serum availability of these elements [44]. The source of organic Se is mushrooms and foods, both of animal (animal red meats, poultry, beef or sheep liver, seafood, eggs, and dairy products) and plant origin. A deficiency of Se, as for other foods, can easily occur in stroke patients who are less likely to consume a variety of beneficial foods, have poorer eating habits, and have an impoverished dietary quality [47]. Magnesium (Mg) maintains cell membrane stability, participates as a cofactor of several enzymes involved in energy metabolism, controls the calcium influx to the neurons as a NMDA receptor blocker, and works as an antioxidant mitigating the effects of oxidative stress [48]. Fruits and vegetables, as well as nuts, seeds, and whole grain products, are the main natural sources of magnesium. However, its absorption is only 30–40% of what is consumed [48]. Inadequate levels of magnesium, zinc, copper, iron, and selenium impair immune competence and the long-term regulation of systemic inflammation [49]. Overall, the improper eating habits that often occur because of the patients’ insufficient food intake contribute to the long-lasting cytotoxic effects of stroke-induced oxidative stress and impair the regulation of inflammation.

### 4.3. Effects of Antioxidant and Anti-Inflammatory Diets: The Mediterranean Diet and Ketogenic Diet

As already mentioned, reducing some of the post-stroke malnutrition-associated consequences is essential so as not to hinder rehabilitation programs. Restoring nutritional deficiencies and re-accustoming the patient to correct eating behavior are an integral part of stroke management. The apparent benefits of the Mediterranean diet (MD) are ascribed to the variety of foods, including aromatic herbs and spices (phytochemicals, vitamins, poly-unsaturated fatty acids, dietary fibers, trace elements, etc.), which, and not individually, work as powerful modulators of oxidative stress and inflammation and endothelial function [27,47]. It is widely known that the consumption of legumes, fruits, vegetables, fish, extra virgin olive oil, fish and nut oils, and leafy vegetables for their content of omega-3 (*n*-3) fatty acids docosahexaenoic acid (DHA) and eicosapentaenoic acid (EPA), polyphenols, vitamins, and minerals could improve neurotransmission and modulate neuronal membrane fluidity, essential for supporting cell signaling and neuronal plasticity [32,35,37,38,39]. Furthermore, adherence to the MD is associated with a high microbiota diversity, with a positive balance between commensal and pathogenic bacteria, and a proper production of neurotransmitters and SCFAs, necessary to ensure the correct functioning of the intestinal barrier [40]. Of interest, re-accustoming the patient to the palatable foods that characterize the core constituent of this eco-sustainable dietary model could contribute to lessening the impairment of taste and smell frequently observed in post-stroke patients.

The hypothesis that the ketogenic diet can supply necessary calories to all body functions shifting the physiologic energetic source from carbohydrate to fat has recently suggested its use, beyond epilepsy and weight loss, as a treatment of neurologic diseases in which the neuron mitochondria are dysfunctional and the impairment of energy metabolism, increased oxidative stress, and inflammation occur, as is the case with stroke [52,53]. Ketone bodies (KBs) comprise acetone, acetoacetate, and β-hydroxybutyrate, and are produced mostly in the liver and in glial cells, namely, astrocytes [53]. Many experimental studies underlined the pleiotropic properties of KBs in animal models and their positive influence in improving or preventing mitochondrial dysfunction as well as in mitigating oxidative and inflammatory damage [88]. There is evidence that KBs can inhibit increased neuronal excitability by modifying pre-synaptic concentrations and the release of the inhibitory γ-aminobutyric acid neurotransmitter (GABA) [54]. A ketogenic diet may also provide neuroprotective effects by improving the mitochondrial function that results in increased mitochondrial energy reserves, with anti-inflammatory effects, together with a Nrf2-induced decrease of ROS generation [88,89]. Accordingly, with the above reported evidence, a ketogenic diet could be an alternative strategy with which to improve rehabilitative outcomes by targeting the crosstalk between oxidative stress and inflammation, particularly burdensome in obese and type II diabetic patients [55].

## 5. Neuronutrition and Stroke-Induced Gut–Brain Axis Disturbance

**Figure 1:**
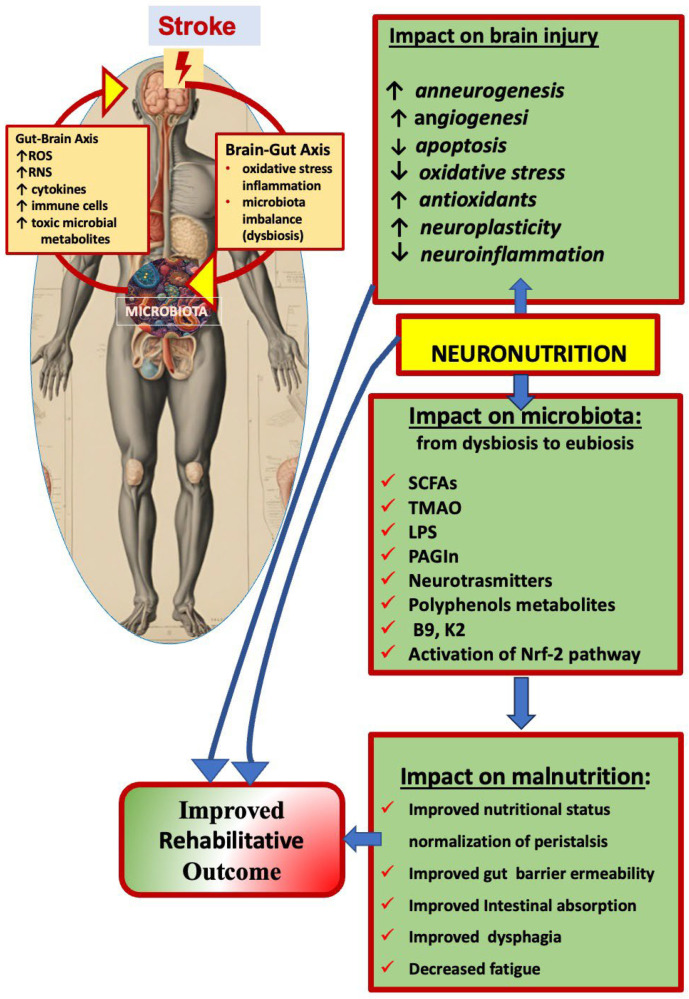
Schematic representation of the impact of neuronutrition on stroke-induced gut–brain axis disturbance.

A schematic representation of the impact of neuronutrition on stroke-induced gut–brain axis disturbance is reported in the Figure 1.

In stroke, the brain–gut axis is altered, and morphological and functional consequences in both directions occur. Stroke-induced oxidative stress and inflammation are involved in the gut dysbiosis and in malnutrition, compromising brain functional repair and rehabilitation effectiveness. Neuronutrition can revert the dysbiosis, positively influence the gut–brain axis signalling, and improve post-stroke recovery by reducing the neuroinflammation oxidative stress, and restoring the microbiota composition improves rehabilitative outcomes.

ROS, reactive oxygen species; RNS, reactive nitrogen species; Nrf-2: nuclear factor erythroid 2-related factor; SCFAs: short-chain fatty acids; BAs: bile acids; TMAO: trimethylamine. *N*-oxide; LPSs: lipopolysaccharides; PAGln: phenylacetylglutamine. ↓ decreasese; ↑ increase.

### 5.1. The Gut–Brain Axis, the Gut Microbiota, and Eubiosis

Articulate bidirectional mechanisms, known as brain-to-gut and gut-to-brain signaling pathways, allow an efficient connection between the brain and the gut and vice versa. In the top-down mechanism, the brain strongly controls the functions of the entire gastrointestinal tract (from peristaltic movements, to the permeability of the intestinal barrier, to the release of neurotransmitters and stress hormones, to the activation of resident immune cells, to the gut microbiome) by means of a multiple signaling system (the vagus nerve, sympathetic prevertebral ganglia, endocrine, immune, humoral links, and gut microbiota) [90]. The gut-to-brain signaling mechanism, also known as the microbiota–gut–brain axis, on the other hand, is, overall, a dynamic system of humoral and nervous afferents to the brain to elicit adequate responses to a vast number of humoral signals coming from the environment. It involves different neural pathways involving vagal and/or spinal afferent fibers, the endocrine system, the hypothalamic–pituitary–adrenal axis, the immune system, and the microbiota and microbiome-derived metabolites [91]. The modulating factor that fulfills a predominant role in gut–brain communication is the gut microbiota, an intestinal ecosystem of a population of fungi, archaea, virus, bacteria, and parasites that, together with a diverse array of microbiome-derived metabolites, interacts with all structures of the intestine to maintain gut homeostasis [91]. The human gut microbiome (1 kg in a healthy and adult subject) is a complex system that resides primarily in the lower gut and lives in a symbiotic relationship with the human host. It consists of approximately more than trillions of microorganisms in a stable ratio themselves. The Firmicutes (F) (e.g., Lactobacillus, Clostridium, and Enterococcus) and Bacteroidetes (B) (e.g., Bacteroides) phyla represent the majority of these intestinal taxonomic categories [92,93,94]. Changes in the abundance of some bacterial taxa were observed in many diseases associated with inflammation [95]. The features of the entire gastrointestinal tract (pH, mucus layer, intestinal redox potential, age of the subject, etc.) and a sensible feedback mechanism cooperating within the microbial community assure a balanced gut microbiota composition, eubiosis, and regulate microbiota growth and stability [93]. Diet and host characteristics including the nutritional status, age, and genetics interfere with the composition of the intestinal microbiota and modify their signaling activity [94,95]. The gut microbiota produces a variety of compounds such as short-chain fatty acids (SCFAs), bile acids (BAs), trimethylamine N-oxide (TMAO), lipopolysaccharides (LPSs), and phenylacetylglutamine (PAGln) [92]. Of note, evidence underlined that the gut microbiota has the capability to produce a range of neurotransmitters as γ-aminobutyric acid, noradrenaline, and dopamine, and that neurotransmitter modulation represents the ways by which the microbiota communicates along the gut–brain axis [94,95,96]. Moreover, efficacious antioxidants, such as bioactive polyphenol-derived metabolites and vitamins such B9 and K2, are also produced by specific microbiota components [50,51]. Interestingly, Lactobacillus spp., a component of the gut microbiota and major group of lactic acid bacteria, play an important antioxidant role by activating the Nrf-2 pathway in response to an excessive ROS/NRS availability in the gut tract, resulting from both the ability of some commensal and pathogenic bacteria to alter their basal generation of mitochondrial ROS, and from the oxidative damage associated with neuroinflammation that characterizes neurodegenerative diseases and ischemic stroke [97,98]. There are multiple and significant factors, independent of pathological conditions, that contribute to shaping the microbiota composition. Dietary eating patterns and the gut microbiota composition are closely connected to each other. Interestingly, a short-term dietary change can alter gut microbial populations within 24 h, although the main enterotypes remain largely unmodified [41,99,100]. Dietary eating patterns (e.g., a high intake of saturated fat and simple sugars) are determinant factors, which are, above all, modifiable, that may modify the biodiversity and representation of taxa with a prevalence of Bacteroidetes, and decreased levels of Firmicutes in humans, as well as preclinical models [101]. On the other hand, the regular consumption of omega-3 fatty acids, found in fatty fish or leafy vegetables, led to increased circulating acids docosahexaenoic acid (DHA) and eicosapentaenoic acid (EPA) that may revert the microbiota composition by stimulating taxonomic groups producing SCFA, thus influencing the gut–brain axis [41]. Macronutrient and fiber contents are important in determining the microbiota composition and its effect on health outcomes and behavior. In addition to the composition of nutrients, the time of food intake and eating patterns have been shown to affect the gut microbiota. The mechanisms through which the microbiota exerts its beneficial or detrimental influences are yet to be better defined, but the elaboration of signaling molecules and the recognition of bacterial epitopes by both intestinal epithelial and mucosal immune cells could be involved [93]. Compelling evidence shows that the gut microbiota influences the nutritional and metabolic homeostasis modulating appetite and food intake, thus preserving a proper nutritional status [5].

### 5.2. Stroke-Induced Modifications of the Gut–Brain Axis and Dysbiosis

Deregulated, the microbiota–gut–brain axis depends on many factors leading to a series of structural and functional consequences, among which, essentially, are the decreased expression of tight junction proteins with a consequent increased intestinal barrier permeability (leaky gut syndrome), increased secretion of pro-inflammatory cytokines, and modified blood–brain barrier permeability with the consequent excessive translocation of immune cells and toxic microbial metabolites into the brain, [91,93,102]. Qualitative and quantitative changes of the microbiota species, each of them with a different catabolism capacity, determine the dysbiosis that is involved in several inflammatory diseases such as obesity, type 2 diabetes, several cardiovascular diseases, neurodegenerative diseases, and ischemic stroke [101,102,103,104]. Evidence suggests that stroke-associated oxidative stress and inflammation can be involved in the destruction of the balanced and physiologic composition of the gut microbiota and can trigger the harmful condition of gut microbiota dysbiosis [5,76,105]. Experimental studies from animal models of stroke showed a significant alteration in the bacterial composition characterized by an excessive growth and amount of the Bacteroidetes phylum and Prevotella genus, together with a significant decrease in the Firmicutes phylum, as well as the Faecalibacterium, Oscillospira, and Lactobacillus genera [106,107,108,109]. Clinical evidence supports the experimental studies and shows that the gut microbiota in patients with stroke was significantly changed in quantitative and qualitative composition [110,111,112]. Dysbiosis in stroke patients consists in an increase of opportunistic pathogens (Streptococcus and Bacteroide) and a decreased number of SCFA-producing bacteria with a consequent decreased plasma concentration of butyrate, essential for the integrity of the intestinal barrier, in the inhibition of the pro-inflammatory cytokine production, and involved in neurogenesis and angiogenesis mechanisms. Of interest, significant associations have been found between the alterations in distinctive gut microbiota and the severity of the neurologic impairment and global functional prognosis [109,110]. Central is also the role of dysbiosis for modifications of appetite and satiety-regulating systems precipitating or worsening malnutrition due to an altered function of the gut–microbiota–brain axis [5,103,104]. Malnutrition and stroke-related symptoms, such as fatigue syndrome, and sarcopaenia, as well as cardio–respiratory–muscle deconditioning are recognized as useful predictors of limited mobility and negatively affect neuronal plasticity and functional recovery, constituting, thus, a real barrier to stroke rehabilitation, namely, physical exercise training [103].

### 5.3. The Gut Microbiota as a Potential Target for Post-Stroke Functional Recovery

Many studies report evidence that the gut microbiota and their metabolites might be considered as an interesting target for post-stroke functional recovery because of their influence on multiple mechanisms via the gut–microbiota–brain axis. Although the relationship between the gut microbiota and post-stroke brain remodeling is yet to be defined, restoring post-stroke dysbiosis is the initial approach for promoting neuroplasticity, stroke recovery, and rehabilitation effectiveness [76,113,114,115]. Findings in animal stroke models show that the normalization of stroke-induced dysbiosis (e.g., a reduction in the levels of Bacteroidetes and an increase in the Proteobacteria and Firmicutes population or SCFA-producing bacteria) mitigates neurological deficits, and improves post-stroke recovery by decreasing inflammation oxidative stress and restoring the SCFA-producing bacteria amount that regulate the levels of BDNF, a well-known neurotrophic factor involved as a keystone molecule in neuroplasticity, learning, and memory [116,117,118,119]. It is known that the recovery of motor function after stroke is mediated by neuroplasticity [120]. Studies on developing rehabilitation strategies aimed at facilitating and maximizing functional outcomes post-stroke showed an emergent role of BDNF as a key facilitator of neuroplasticity involved in motor learning and rehabilitation after stroke [121]. Experimental findings showed that probiotic or prebiotic supplementation improves the microbiota composition that, in turn, increases BDNF levels in various brain regions [2,122]. Their role as inflammatory modulators has been well-evidenced in experimental studies [123]. Evidence demonstrated that the immune modulation performed by probiotics might be due to the release in the gut of the anti-inflammatory cytokine [120]. Therefore, the regular intake of probiotics, especially in malnourished stroke patients, might be useful for maintaining the homeostasis of the gut microbiota, and then the proper working of the gut–brain axis, by preventing the destruction of tight junction proteins and inhibiting pathogenic bacterial overgrowth and the consequent infections. Among the natural compounds enhancing neuroplasticity, polyphenols, a wide family of molecules present in many food sources (vegetables, red fruits, green tea, coffee, and wine), show neuroprotective properties such as strong antioxidants and anti-inflammatory molecules [27,60]. However, the beneficial effectiveness of these compounds for the brain repair mechanisms after a stroke is strictly dependent on the amount consumed and their effective availability, via the systemic circulation, on the neurovascular units adjacent to the ischemic lesion. Further investigation and controlled clinical studies are necessary in order to validate their therapeutic efficacy.

## 6. Conclusions

Stroke-related long-term sequelae and their high social and economic burden require effective and affordable measures, improving brain functional recovery and the effectiveness of rehabilitative treatment. The recognition of the role of neuronutrition as an adjuvant strategy for integrating the brain recovery processes emerges from experimental research findings that have demonstrated how the properties of some nutrients and dietary patterns can contribute to influencing the post-stroke recovery processes by interfering with oxidative stress, inflammation, and gut–brain axis disturbance. Limited information is still available on how experimental results might translate to the recovery of patients after stroke. Rigorous studies in prospective clinical trials are therefore required in order to verify whether the experimental nutritional interventions on animal models or in vitro research can find clinical applications. Of note, the complexity of human nutritional intervention makes it difficult to attribute a role/function to isolated dietary components by extrapolating them from the context of the multiple interactions occurring between the different components of a single food and, even more, of the entire dietary pattern. The improvements in the technology and implementation of omics sciences in terms of nutrition and rehabilitation allow us to apply personalized nutrition-based care and a personalized approach to rehabilitation. Indeed, the simultaneous measurement of an enormous number of biomolecules that can “capture” many potential biological factors that contribute to the high inter-individual variability of the stroke recovery course and rehabilitation outcome will be an effective methodologic setting for better results. Advances in our understanding of the mechanisms underlying the impact of nutrition on the brain and the microbiota–gut–brain axis will facilitate the real development of neuronutritional interventions and their clinical application to optimizing post-stroke functional recovery.

## Figures and Tables

**Table 1:** Dietary sources of nutrients with potential influence on the mechanisms involved on post-stroke recovery and rehabilitation effectiveness.

Food Sources	Bioactive Substances	Specific Effects	References
Legumes, fruits, vegetables, fish, extra virgin olive oil, fish and nut oils, and leafy vegetables	Omega-3 fatty acids: EPA, DHA, and DPA	Improved neurotransmission, neuronal membrane fluidity, cell signalling, neuronal plasticity, BDNF, and microbiota composition (taxonomic groups producing SCFA); improved rehabilitation outcomes	[5,28,29,31,35,37,38,39,40,41]
Vegetables, green tea, coffee, wine, extra virgin olive oil, and well-balanced diet (the Mediterranean diet, MD)	Polyphenols	Antioxidant and anti-inflammatory properties; neuroprotection; scavenging free radicals, chelating metals, dampening pro-oxidative enzymes	[27,32,35,37,38,39,42,43]
Fruits, legumes, animal red meats, poultry, beef or sheep liver, seafood, eggs, herbs, spices, high dietary fiber intake, nuts, seeds, and whole grain products	Minerals (Zn, Mg, Se, and Cu)	Co-factors of antioxidant enzyme, long-term regulation of systemic inflammation, and metabolic homeostasis	[24,25,26,27,43,44,45,46,47,48,49]
Well-balanced diet, vegetables, fruits, dairy products, egg yolk, and offal and liver of pigs, sheep, and cattle.	Vitamins (A, C, E, D, B group, and K2)	Antioxidants and anti-inflammatory; anti-apoptosis; and neurorestorative (post-stroke recovery, rehabilitation effectiveness)	[27,44,47,50,51]
Diet with high fat, moderate protein content, and low carbohydrates (ketogenic diet)	Ketone bodies (acetone, and β hydroxybutyrate acetoacetate)	Prevention of mitochondrial dysfunction, decreased oxidative and inflammatory damage, and GABA release	[52,53,54,55]
Fruits, legumes, and vegetables	dietary fiber (carbohydrate polymers, and non-digestible carbohydrates)	Gut microbiome composition, intestinal peristalsis, acid–base balance, and decreased proinflammatory cytokines	[5,27,47]
